# Redox State and Mitochondrial Respiratory Chain Function in Skeletal Muscle of LGMD2A Patients

**DOI:** 10.1371/journal.pone.0102549

**Published:** 2014-07-31

**Authors:** Mats I. Nilsson, Lauren G. Macneil, Yu Kitaoka, Fatimah Alqarni, Rahul Suri, Mahmood Akhtar, Maria E. Haikalis, Pavneet Dhaliwal, Munim Saeed, Mark A. Tarnopolsky

**Affiliations:** Department of Pediatrics and Medicine, Neuromuscular Clinic, McMaster University Hospital, Hamilton, Ontario, Canada; University of alabama at birmingham, United States of America

## Abstract

**Background:**

Calpain-3 deficiency causes oxidative and nitrosative stress-induced damage in skeletal muscle of LGMD2A patients, but mitochondrial respiratory chain function and anti-oxidant levels have not been systematically assessed in this clinical population previously.

**Methods:**

We identified 14 patients with phenotypes consistent with LGMD2A and performed *CAPN3* gene sequencing, CAPN3 expression/autolysis measurements, and *in* *silico* predictions of pathogenicity. Oxidative damage, anti-oxidant capacity, and mitochondrial enzyme activities were determined in a subset of muscle biopsies.

**Results:**

Twenty-one disease-causing variants were detected along the entire *CAPN3* gene, five of which were novel (c.338 T>C, c.500 T>C, c.1525-1 G>T, c.2115+4 T>G, c.2366 T>A). Protein- and mRNA-based tests confirmed *in* *silico* predictions and the clinical diagnosis in 75% of patients. Reductions in antioxidant defense mechanisms (SOD-1 and NRF-2, but not SOD-2), coupled with increased lipid peroxidation and protein ubiquitination, were observed in calpain-3 deficient muscle, indicating a redox imbalance primarily affecting non-mitochondrial compartments. Although ATP synthase levels were significantly lower in LGMD2A patients, citrate synthase, cytochrome *c* oxidase, and complex I+III activities were not different from controls.

**Conclusions:**

Despite significant oxidative damage and redox imbalance in cytosolic/myofibrillar compartments, mitochondrial respiratory chain function is largely maintained in skeletal muscle of LGMD2A patients.

## Introduction

Limb-girdle muscular dystrophy (LGMD) are a heterogeneous group of genetic disorders and characterized by progressive weakness and wasting of the proximal limb girdle muscles and dystrophic muscle pathology. To date, 24 forms of LGMD have been identified with either autosomal dominant (1A-1H) or recessive (2A-2Q) inheritance patterns. Primary calpainopathy (LGMD2A, OMIM 253600), caused by mutations in the 40-kb *CAPN3* gene (OMIM 114240, mapped to 15q15.1-q21.1), is the most frequent form of recessive LGMD, with a prevalence of 1∶15,000–1∶150,000 depending on the population [1]. Disease onset generally occurs in early childhood to the second decade of life and is distinguished by an increase in serum creatine kinase (CK), symmetrical involvement of shoulder/pelvic girdles, and a dystrophic muscle pathology [2], ultimately causing loss of ambulation and wheel-chair dependence in adulthood [3].

The *CAPN3* gene contains 24 exons encoding for the 94-kDa Na^+^/Ca^2+^-dependent cysteine protease calpain-3 (CAPN3) [4], [5], which serves both structural and proteolytic roles within sarcomeres of skeletal muscle [6], [7], [8], interacting mainly with α-actinin and titin to regulate myofibrillar disassembly, protein turnover, and mechanotransduction [9], [10], [11]. Muscle-specific CAPN3 consists of four domains (I–IV [Fig. S1]) and has three unique insertions sequences NS, IS1 (autolytic sites) and IS2 (titin-binding and nuclear translocation signal). Deficiency of CAPN3 is associated with build-up of toxic debris, oxidative damage, degeneration, and necrosis in LGMD2 patients, proving that it is indispensable for the maintenance and function of skeletal muscle. While the majority of the reported ∼480 pathological *CAPN3* mutations impair autolysis and enzyme activity of calpain-3 (www.dmd.nl), titin-anchorage and substrate binding may also be affected [3], which is exemplified by the fact that 20–30% of LGMD2A patients exhibit normal calpain-3 protein levels and no loss in autolytic activity [12], [13], [14], [15]. As such, the assessment of protein expression and Ca^2+^-dependent autolysis of calpain-3 may be a cost-effective diagnostic approach (particularly in the absence of substrate-specific enzyme activity assays); however, *CAPN3* gene sequencing is essential for assigning a specific diagnosis in a significant portion of LGMD2A patients and remains the gold standard. Gene sequencing allows the practitioner to differentiate between primary and secondary calpainopathies and to perform genotype-phenotype correlations. Furthermore, the discovery of novel mutations improves our current understanding of molecular pathology, protein function, and may uncover previously unknown cellular roles of calpain-3.

Among the recently elucidated roles of CAPN3 is stabilization of the ryanodine receptor (RyR) and regulation of Ca^2+^ release during excitation-contraction coupling in the skeletal muscle triads. RyR expression, Ca^2+^ release, and CAMKII signaling are significantly reduced in CAPN3 knock-out (KO) mice, causing a decreased sensitivity to exercise stimuli, abnormal morphology and organization of mitochondria, decreased ATP production, and preferential involvement of slow-twitch muscle fibers [6], [7], [8], [16]. Although proteomic studies support the notion that CAPN3 is an important regulator of mitochondrial function [16], [17], and slow-twitch muscle fibers (↑ mitochondrial abundance in slow vs. fast) are predominately affected in LGMD2A patients [7], [18], [19], expression and activities of rate-limiting enzymes in the Kreb’s cycle and mitochondrial respiratory chain (MRC) have not been assessed in calpain-3 deficient humans to date. Furthermore, the effect of calpainopathy on mitochondrial anti-oxidants are unknown despite the fact that oxidative damage is a hallmark of LGMD2A and mitochondria are postulated to generate the majority of cellular free radicals [20]. To this end, we measured oxidative damage, anti-oxidant capacity, protein ubiquitination, and expression/activities of rate-limiting mitochondrial enzymes in skeletal muscle of genetically confirmed LGMD2A patients. From our cohort of 14 subjects, we identified 21 mutations of the *CAPN3* gene, including five previously unreported sequence variants, and present biochemical data and *in* *silico* predictions of pathogenicity to strengthen genotype-phenotype correlations.

## Methods

### Clinical diagnosis, muscle biopsy and blood collection

All experiments were conducted according to the principles expressed in the Declaration of Helsinki. Written informed consent for muscle biopsies and approval for the use of archived patient samples were given by all subjects and the research ethics board at Hamilton Health Sciences (HIREB project # 10-327-T).

Fourteen out of 272 patients with confirmed or suspected muscular dystrophy in the Neuromuscular Disease Clinic at McMaster University Hospital exhibited clinical features consistent with LGMD2A. Major diagnostic criteria included absence of an autosomal dominant or X-linked inheritance pattern, atrophy and progressive weakness of shoulder/hip girdles, preferential posterior involvement of thighs/calves, sparing of facial, oculomotor, and cardiac muscles, and CK levels 5–80 times above normal [2], [21], [22]. Additional clinical features seen in some patients were waddling gate, winging scapulae, loss of ambulation, and wheelchair dependence. Upon review of clinical records, patients were further classified into Erb phenotype (scapulohumeral), early pelvifemoral phenotype (≤12 y), classical pelvifemoral phenotype (Leyden-Möbius;13–29 y), late onset pelvifemoral phenotype (≥30 y), or asymptomatic phenotype (hyperCKemia) if possible [13], [21]. To confirm clinical diagnosis, whole blood from antecubital vein (N = 14) and/or a skeletal muscle biopsy from *vastus lateralis* (N = 12) were obtained and sent for *CAPN3* mutational testing and electron/light microscopy. Upon receiving genetic and pathology results, a sub-set of biopsies, previously snap-frozen in liquid nitrogen and stored at −80°C, were used for mitochondrial and CAPN3 mRNA/protein expression studies (WB and RT-PCR), oxidative damage/autolytic activity tests, and immunohistochemistry. For diagnostic purposes, those patients with one identified *CAPN3* sequence variant were prioritized for biochemical analyses and compared to subjects with proven pathology and age/gender-matched controls. Control muscles were obtained from our biopsy bank, which contains specimens from healthy subjects that have previously consented to the use of their tissues in our research. Bioinformatic software tools were used to predict pathogenicity of all mutations and strengthen genotype-phenotype correlations. Molecular tests and/or gene sequencing were done to rule out Duchenne/Becker muscular dystrophy (DMD/BMD), facioscapulohumeral muscular dystrophy (FSHD), Emery Dreifuss muscular dystrophy, LGMD2B (*DYSF*), LGMD2C-F (sarcoglycanopathies), and LGMD2I (*FKRP*) in select patients.

### *CAPN3* mutation and *in* *silico* analyses

Whole blood samples from patients were sent to PreventionGenetics (Marshfield, WI) and Athena Diagnostics (Worcester, MA) for *CAPN3* DNA sequence testing as previously described [23], [24]. PreventionGenetics extracted genomic DNA using a Gentra PUREGENE kit and used PCR to amplify the full coding region (24 exons, 2466 basepairs) as well as ∼50 bases of flanking intronic or other non-coding sequences. After cleaning of PCR products, cycle sequencing was carried out using the ABI Big Dye Terminator v.3.0 kit. Products were resolved by electrophoresis on an ABI 3130×l capillary sequencer and compared with reference sequences. Sequencing was performed separately in both forward and reverse directions. Similarly, Athena Diagnostics isolated highly purified genomic DNA, followed by automated uni-directional DNA sequencing of the coding region and 20 bases surrounding each exon. All abnormal variants were confirmed bi-directionally and detectable at an overall sensitivity approaching 99%. All test results were reviewed, interpreted, and reported by ABMG certified clinical molecular geneticists.

Pathogenicity of identified mutations was predicted using bioinformatics software as previously described by our laboratory [23], [25]. Missense mutations were assessed by SIFT (sift.jcvi.org, SIFT sequence, UniProt-TrEMBL 2009 Mar) and PolyPhen2 (http://genetics.bwh.harvard.edu/pph2/) whereas intronic sequence variants were evaluated by the Human Splicing Finder program, version 2.4.1 (http://www.umd.be/HSF/). NetGene2 and BDGP Splite Site Prediction were used to verify the results obtained from HSF. Evolutionary conservation analysis of non-mutated nucleotides/amino acids was carried out using the UCSC Genome Browser (http://genome.ucsc.edu/cgi-bin/hgGateway). Mutations that alter highly conserved nucleotides/amino acids are likely to be deleterious, as these nucleotides/amino acids may be vital to mRNA/protein structure or function. As such, evolutionary conservation analysis was carried out with the UCSC Genome Browser using the “Feb.2009 (GRCH37/hg19)” assembly. Conservation of non-mutated nucleotides/amino acids across 46 species from the subphylum *Vertebrata* was assessed, including 36 mammalian species (*Mammalia;* 33 eutharians with chorioallontoic placenta, two metatherians with choriovitelline/yolk placenta, and one egg-laying prototherian), five species of bony fish (*Osteichthyes*), one jawless fish (*Agnatha*), two birds (*Aves*), one frog (*Amphibia*), and one lizard (*Reptilia*). Among these vertebrates were chimp, orangutan, rhesus, mouse, rat, guinea pig, rabbit, dolphin, cow, dog, elephant, opossum, lizard, *Xenopus tropicalis*, tetraodon, fugu, zebrafish, and lampreys. Mutation nomenclature adheres to the guidelines from the Human Genome Variation Society and nucleotide accession # NM000070.2 and protein accession # NP000061.1.

### RNA isolation and quantitative real-time PCR (RT-PCR)

Total RNA isolation, first strand cDNA synthesis, and PCR amplification were performed as previously described with minor modifications [23]. Total RNA was extracted from approximately 25 mg of quadriceps muscle that was homogenized in 1 mL TRIzol® reagent (Invitrogen, Burlington, ON, Canada). Following homogenization, 0.2 mL of chloroform was added to each sample and the clear aqueous phase was transferred to RNeasy spin columns, followed by RNA isolation as per manufacturer’s instructions (Qiagen, Germantown, Maryland, USA). The samples were DNase treated on the spin columns to prevent contamination with genomic DNA (Qiagen) and the absorbances at 230 nm (OD_230_), 260 nm (OD_260_), and 280 nm (OD_280_) were determined using an ultraviolet spectrophotometer (NanoDrop 1000; Thermo Scientific). Measurements were repeated twice with a coefficient of variation of <7.5% and OD_260/280_ and OD_260/230_ ratios >1.5. Reverse transcription was performed on 150 ng of total RNA primed with random hexamers on a gradient thermocycler (MyCycler, Biorad, Hercules, CA, USA) as per manufacturer’s instructions (Applied Biosystems, Foster City, CA, USA). Real-time polymerase chain reaction (RT-PCR) was performed with the 7300 Real-time PCR system (Applied Biosystems, Foster City, CA, USA) using SYBR® Green chemistry (PerfeCTa SYBR® Green Supermix with ROX, Quanta Biosciences, Gaithersburg, MD, USA) with standard thermocycling conditions. Primers, based on human gene sequences from GenBank (http://www.ncbi.nlm.nih.gov/genbank/), were designed for CAPN3 (NM_000070; F: 5′-atgactcggaggtgatttgc-3′, R: 3′-tttgggaacctcgtagatgg-5′) and β2-microglobulin (NM_004048.2; F: 5′-ggctatccagcgtactccaa-3′; R: 3′-gatgaaacccagacacatagca-5′) using Primer3 software (http://biotools.umassmed.edu/bioapps/primer3_www.cgi). Melt curve analysis was performed on all primers to ensure the validity of amplification. Tm for forward and reverse CAPN3 primers was 60.08°C and 59.93°C, respectively. CAPN3 mRNA expression was thereafter quantified using the 2^−ΔCt^ method and normalized to β2-microglobulin.

### Western blotting

In order to minimize CAPN3 Ca^2+^ autolysis, whole quadriceps muscle was homogenized in 1∶10 wt/vol in 0.4 M Tris hydrochloride, 25 mM EGTA ([Ca^2+^] <10 nM), 4% SDS, and protease inhibitor mixture (Complete Tablets, Roche) (pH 6.8) as previously described with minor modifications [11], [26]. Immediately following homogenization, samples were heated to 95°C in SDS loading buffer for 10 min (0.125 M Tris hydrochloride, 10% glycerol, 4% SDS, 4 M urea, 10% mercaptoethanol, and 0.0001% bromophenol blue, pH 6.8) and stored at −80°C. A small aliquot of muscle homogenate, obtained prior to denaturation, was analyzed for protein concentration by a standard colorimetric assay [27].

The samples were loaded in equal amounts on 4–15% SDS polyacrylamide gels (10–20 µg), electrophoresed for one hour at 25 mA, and wet-transferred in Otter buffer (49.6 mM Tris, 384 mM glycine, 20% methanol, and 0.01% SDS) for 50 min at 400 mA onto 0.45 µM nitrocellulose membranes (Amersham Hybond-ECL, GE Healthcare). Following a 1-hr block step, membranes were incubated at 1∶1000 (unless otherwise noted) with primary antibodies from Novacastra (CAPN 3*, NL-CALP-12A2, 1∶100), Abcam (4-HNE^#^, ab48506, 1∶3000; SOD1*, ab16831; SOD2*, ab13534), MitoSciences (total OXPHOS*, MS601), and Santa Cruz (Ub*, sc-8017; NRF-2*, C-20/sc-722; Keap-1*, sc15246) at 1∶1000 overnight at 4°C. Standard HRP-linked secondary antibodies from GE Healthcare (NA931V [anti-mouse]; NA934V [anti-rabbit]) and Bio-Rad (anti-goat) diluted 1∶5000 were used to detect primary IgGs, and reacted with Immobilon Western Chemiluminescent HRP Substrate from Millipore. Lastly, the membranes were developed in a dark room with Amersham Hyperfilm (GE Healthcare), and relative intensities of specific antigen bands or entire lanes (ubiquitin and 4-HNE) were quantified digitally with ImageJ 1.37v software. Blocking was performed in 1% milk^*^ (m) or 5% m^#^ (1×TBS), primary incubations in 1% m^*^ or 5% BSA^#^ (1×TBS), and secondary incubations in 1% m^*^ or 5% m^#^ (1×TBS). All incubation steps were followed by 3×5 min washes in TBS. CAPN3 blots were repeated twice with homogenates prepared from two separate pieces of muscle. Equal loading was verified by Ponceau S stain following wet transfer, which in our hands is a more consistent way of normalizing WB data compared to the use of single house-keeping proteins. Select membranes were probed for actin to confirm accuracy of Ponceau S.

### Mitochondrial enzyme assays

Cytochrome *c* oxidase (COX; EC 1.9.3.1), complex I+III, and citrate synthase (CS; EC 2.3.3.1) activities were measured in quadriceps homogenates as previously described by our group [28], [29], [30]. All samples were analyzed in duplicate on a Cary 300 Bio UV–visible spectrophotometer (Varion, Inc., Palo Alto, CA) and the intra-assay coefficient of variation for all samples was less than 5%.

For COX activity, oxidized cytochrome *c* (Sigma C7752) was reduced by sodium ascorbate in 0.05 M potassium phosphate buffer (KH_2_PO_4_, pH 7.4). Fifteen microliters of muscle homogenate were added to 955 µL of 0.05 M potassium phosphate buffer and 15 µL of reduced cytochrome *c* in a 1.5 mL cuvette. The rate of oxidation of reduced cytochrome *c* was measured at 550 nm for 3 min at 37°C. COX activity was expressed in nmol/min/mg protein.

Complex I+III activity (NADH-cytochrome *c* oxidoreductase) was assessed by adding 15 µL of oxidized cytochrome *c* (40 mg/ml) to 1 mL of reaction buffer (0.1 M potassium phosphate and 1 mM sodium azide, pH∼7.0, 37.5°C) in two 1.5 mL cuvettes. Following mixing, five µL of 1 mM rotenone (Sigma R8875) was added to a “rotenone-sensitive” cuvette (back) and 20 µl of muscle homogenate (∼50–150 µg total protein) were added to both (front and back). A blank reading was done after 1 min equilibration and 5 µL of NADH was added to the front cuvette only at the start of the measurement. The reduction of oxidized cytochrome *c* was measured over two minutes at 550 nm. Activity was expressed in µmol cytochrome *c* reduced ⋅ min^−1^⋅ mg protein^−1^.

CS activity was determined by measuring the formation of thionitrobenzoate anion. Fifteen microliters of muscle homogenate were added to 810 µL buffer (0.1 M Tris–HCl buffer, pH 8.0) along with 10 µL of acetyl CoA (7.5 mM in 0.1 M Tris-HCL buffer, pH 8.0) and 100 µL of 0.1 mM dithionitrobenzoic acid. The reaction was started by adding 50 µL of 9.0 mM oxaloacetate. Absorbance was measured at 412 nm for 2 min at 37°C. CS activity was expressed in nmol·min^−1^·mg of protein^−1^.

### Calpain 3 autolytic activity immunoblots

The autolytic activity assay is based on the observation that full-length CAPN3 (94-kDa) undergoes gradual degradation into lower molecular weight products (56-, 58-, and 60-kDA [NL-CALP-12A2]) when incubated in a calcium-enriched saline solution for ≥5 min [14], [15], [31]. Cellular Ca^2+^ concentrations are ∼100 nM at rest and increase to ∼250 nM for 24 hrs following eccentric exercise, which is enough to activate CAPN3 autolysis [11]. As such, muscle was homogenized in 1∶10 wt/vol saline solution (0.9% NaCl) with 5 mM CaCl_2_ and incubated at room temperature for 5 min to ensure partial autolysis. The reaction was blocked by adding 1∶1 vol/vol loading buffer containing Ca^2+^-chelating EDTA (0.05 DTT, 0.1 M EDTA, 0.125 M Tris hydrochloride, 4% SDS, 10% glycerol, and 0.005% bromophenol blue, pH 8.0), followed by immediate heating at 95°C for 10 min and storage at −80°C. Protein concentration and CAPN3 expression were measured as previously described for the conventional Western blot. CAPN3 autolytic tests were repeated twice with homogenates prepared from two separate pieces of muscle. Equal loading was verified with Ponceau S stain as previously described.

### Calpain 3 immunohistochemistry

Quadriceps muscle (1.5 mm^3^) was embedded in Optimal Cutting Temperature compound (OCT) and immersed in liquid nitrogen-cooled isopentane, followed by cryosectioning of 8 µm slices that were mounted on polylysine-coated slides and stored at −80°C. The slides were brought to room temperature, fixed in acetone for 10 min, rinsed 2×2 min, and stained according to manufacturer’s protocol using the Vector Elite ABC detection system (Vector Laboratories, Burlingame, CA) [32]. Briefly, slides were incubated with 1∶50 CAPN3 (NL-CALP-12A2) for 60 min, followed by quenching of endogenous peroxidase activity with 0.3% H_2_O_2_ and washing 3×2 min_._ A secondary biotinylated anti-mouse antibody was applied at a 1∶500 dilution for 60 min and samples washed for 3×5 min. Slides were then incubated with Vectastain ABC reagent for 30 min, washed 1×5 min, and exposed to peroxidase substrate (3,3′-diaminobenzidine; DAB) for 5 min. Following a rinse in tap-water, the sections were counterstained with hematoxylin for nuclear detail, dehydrated in ascending concentrations of ethanol, and cleared in xylene to increase tissue transparency and improve focus of internal structures. Sections were cover-slipped with Permount and visualized using bright-field microscopy at 400× total magnification as previously described [29]. Antibody incubations and wash steps were done in 0.25% BSA/0.025% Triton X100/TBS solution and TBS, respectively.

### Statistical analysis

Data analysis was performed using independent t-tests (Sigma Stat®, ver. 3.5). Statistical significance was set at P≤0.05. To increase reproducibility, assays were repeated twice for each patient and presented as means ± standard error.

## Results

### Clinical findings

We studied 14 patients (10 males and 4 females [Table 1]) that fulfilled the diagnostic criteria for LGMD2A proposed by the European Neuromuscular Centre Workshop [22], including atrophy and progressive weakness of shoulder/hip girdles, elevated CK levels, and muscle biopsies consistent with a dystrophic/myopathic process. The age at onset ranged from 4 to 39 y and the majority of patients presented with pelvic girdle weakness or asymptomatic hyperCKemia (92.9%). In this cohort we identified two subjects with early onset (14.3%), six with the classical Leyden Mbius phenotype (42.9%), four with late onset (28.6%), one asymptomatic (7.1%), and one unspecified (7.1%). Surprisingly, no patients exhibited primary shoulder-girdle involvement/onset (Erb phenotype). At the time of the investigation three subjects were wheelchair-bound (P5, P6, and P8) and one (P4) had passed away.

**Table 1:** LGMD2A phenotypes, CAPN3 sequence variants, and *in* *silico* predictions.

ID	AAge	Mutation	CEx/In	Nucleotide	Amino Acid	DDom	EZyg	FNov	GBioinformatics		
Sex	BPheno								(missense/deletion)		
									SIFT	Poly2	Cons
* **P1**	37/31/14	Transition	10	c.1250 C>T	p.Thr417Met	III	Het	N	Y	Y	42
**M**	Classical	Transition	4	c.500 T>C	p.Phe167Ser	II	Het	Y	Y	Y	44
* **P2**	39/38/34	Transversion	22	c.2338 G>C	p.Asp780His	IV	Het	N	Y	Y	42
**M**	Late	Transversion	22	c.2366 T>A	p.Leu789Gln	IV	Het	Y	Y	Y	43
* **P3**	39/36/36	Deletion	13	c.1573_1575 del	p. Phe525del	III	Het	N	–	–	37
**F**	Asympt.	Transition	20	c.2120 A>G	p.Asp707Gly	IV	Het	N	Y	Y	41
* **P4**	^†^56/53/18	Transversion	19i	IVS19+4 T>G	Cryptic splice site$	IV	Hom	Y	–	–	–
**M**	Classical	(intronic)		(c.2115+4 T>G)							
**P5**	w28/23/14	Transversion	4i	IVS4+1 G>C	Donor splice site**^#^**	II	Hom	N	–	–	–
**M**	Classical	(intronic)		(c.632+1 G>C)							
**P6**	w39/32/4	Deletion	4	c.550 del A	p.Thr184ArgfsX36	II	Hom	N	–	–	–
**F**	Early	(Frameshift)									
**P7**	54/50/39	Transition	11	c.1477 C>T	p.Arg493Trp	III	Het	N	Y	Y	40
**F**	Late	Deletion (Frameshift)	4	c.550 del A	p.Thr184ArgfsX36	II	Het	N	–	–	–
**P8**	w37/33/15	Deletion	3	c.483 del G	p.Ile162SerfsX17	II	Hom	N	–	–	–
**M**	Classical	(Frameshift)									
**P9**	15/14/13	Transition	9	c.1162 C>T	p.Gln388Stop	II	Het	N	–	–	–
**M**	Classical	Transition	11	c.1465 C>T	p.Arg489Trp	III	Het	N	Y	Y	38
* **P10**	54/50/c	Transition	13	c.1621 C>T	p.Arg541Trp	III	Het	N	Y	Y	38
**M**	Early	Transition	2	c.338 T >C	p.Ile113Thr	II	Het	Y	Y	N	33
* **P11**	40/33/30	Transversion	10	c.1257 T>G	p.Asp419Glu	III	Het	N	Y	Y	43
**M**	Late										
* **P12**	39/32/27	Transversion	11i	IVS11–1 G>T	Acceptor splice	III	Het	Y	–	–	–
**F**	Unspecified	(intronic)		(c.1525–1 G>T)	site‡						
* **P13**	45/45/38	Transition	1	c.224 A>G	p.Tyr75Cys	I	Het	N	Y	Y	35
**M**	Late	Transition	8	c.1099 G>A	p.Gly367Ser	II	Het	N	Y	Y	43
**P14**	30/28/15	Transition	4	c.527 T>C	p.Val176Ala	II	Het	N	Y	Y	43
**M**	Classical	Deletion	5	c.759_761 del	p. Lys254 del	II	Het	N	–	–	41

A Current age/age at biopsy (or genetic confirmation)/first symptoms noted, ^†^ = deceased, w = wheelchair, c = childhood;

B Phenotypic classification into Erb (scapulohumeral), early pelvifemoral (≤12 y), classical pelvifemoral (Leyden-Möbius;13–29 y), late onset pelvifemoral (≥30 y), or asymptomatic phenotype (hyperCKemia);

C *CAPN3* exon/intron;

D CAPN3 domains;

E Zygosity;

F Novelty;

G *In silico* predictions of pathogenicity of missense mutations with SIFT (Uni-Prot TrEMBL 2009 Mar) and PolyPhen-2 (v2.2.2r398). Y = pathological; N = benign. Conservation of exchanged/deleted amino acids with UCSC genome browser (Human Feb. 2009 [GRCh37/hg19] Assembly) across 46 species. Effects of intronic mutations on pre-mRNA splicing with Human Splicing Finder program (version 2.4.1).

$ Mutation in 5′ end of intron 19 creates a cryptic splice site that is used instead of the regular site.

#Mutation in 5′ end of intron 4 destroys donor splice site.

‡ Mutation in 3′ end of intron 11 destroys the acceptor splice site.

*Selected for further biochemical testing.

### Pathological findings

Biopsies and/or blood were collected and used for *CAPN3* mutational analysis and muscle histopathology as described in the methods section (mean age 35.6±2.9 y, range 14–53 y) [Table 1, Figs. S1–S2]. For further diagnostic and biochemical testing, a sub-set of patients were selected (N = 8; mean age 39.8±3.0 y, range 31–50 y) and compared to age- and gender-matched controls (N = 7; mean age 36.6±3.7 y, range 25–50 y) or as noted in the figure legends. Muscle histopathology (electron microscopy and IHC) ranged from mild to moderate to acute dystrophic processes and consisted of internalized nuclei, variation in fiber size, necrosis, degeneration/regeneration, and fiber splitting. Three patients (P1, P10, and P11) were randomly chosen for fiber-type quantification and revealed selective type 1 atrophy (−16% avg. diameter vs. CON) and type II dominance in P11 (83% fast/17% slow vs. 61%/39% in CON) [Fig. S2]. PAS, Oil-Red-O, succinate dehydrogense, cytochrome *c* oxidase, and NADH dehydrogenaze stains generally showed normal triglyceride abundance, glycogen content, and activity/distribution of mitochondria in all patients. Intra-mitochondrial crystalloid inclusions were present in electron micrographs of P4, but no other mitochondrial abnormalities were observed. Unless otherwise noted, immunohistochemistry showed normal reactivity towards dystrophin, spectrin, merosin, sarcoglycans (α,β,γ,δ), beta dystroglycan, caveolin III, desmin, and dysferlin in all subjects.

### Genotype-phenotype correlations

**Figure 1:**
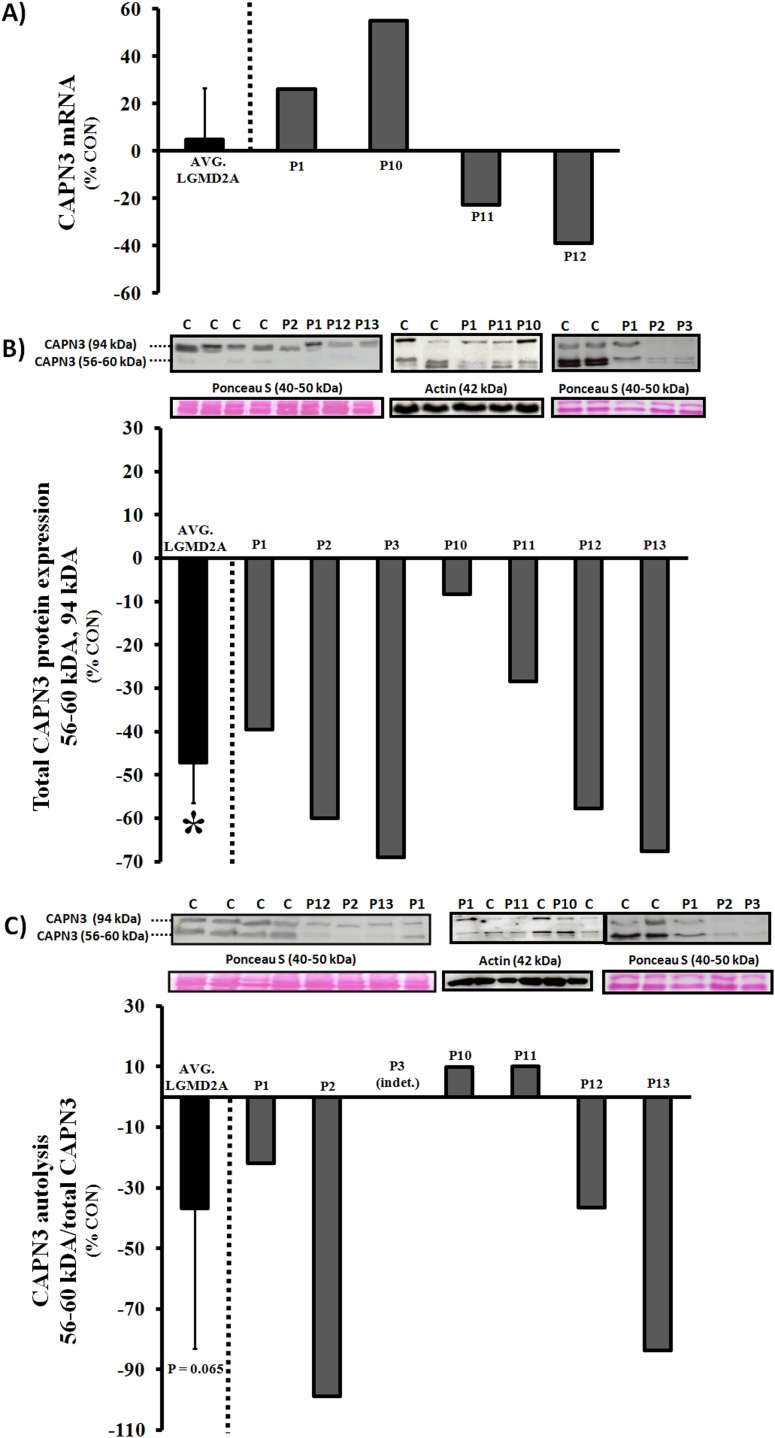
Expression and autolysis of CAPN3 in quadriceps muscle of LGMD2A patients. A) CAPN3 mRNA expression (RT-PCR); B) CAPN3 protein (Western blot); C) Ca^2+^-induced autolytic activity of CAPN3 (Western blot). Calpain-3 autolytic activity is presented as a ratio between autolyzed fragments and total CAPN3 (representing *relative autolytic capacity* of protein present), but the results should also be interpreted in the context of total protein expression from regular Western blots. Logically, a reduction in total CAPN3, obtained from Ca^2+^-free homogenates, equates to a decrease in *total autolytic capacity*. All CAPN3 data were normalized to a suitable housekeeping gene (β2-microglobulin), protein (actin), or total proteins levels (Ponceau S stain) and graphed as % age/gender-matched healthy controls. Representative images of CAPN3 blots (56–60 kDa, 94 kDa), Ponceau S stain (40–50 kDa), or actin (42 kDa) are shown in B–C. For total protein expression and autolytic activity, N = 7 (LGMD2A) and N = 8 (control), while N = 4 (LGMD2A) and N = 6 (control) for mRNA. Bars to the far left represent the average of all LGMD2A patients (AVG. LGMD2A). INDT: Indeterminate. *Significantly lower compared to controls (P≤0.05).

Gene sequencing of the *CAPN3* gene revealed 21 sequence variants in our cohort of 14 patients, including 13 missense mutations, 1 nonsense mutation, 4 deletions (frameshifts/premature stop), and 3 intronic mutations (Table 1). Consistent with idea of mutation clustering in specific exons of the *CAPN3* gene (i.e. 1, 4, 5, 8, 10, 11, 21), ∼60% of identified variants in this study occurred in aforementioned exons (Fig. S1). The majority of patients were compound heterozygotes with the exception of four homozygous individuals (P4, P5, P6, and P8) and two subjects with one mutation identified but with a clinical and/or biochemical phenotype consistent with LGMD2A (P11 and P12). All mutations were predicted to be pathogenic with bioinformatic software programs, exchanging highly conserved amino acids in the enzyme or nucleotides indispensable for splicing of the pre-mRNA. Five of these pathological sequence variants were novel and had not been reported in the Leiden database previously. Biochemical analyses were a priority in those patients exhibiting novel mutations (P1, P2, P4, P10, and P12) and/or one pathological sequence variant (P10 and P11), which were compared to subjects with proven pathology (P3 and P13) and age/gender-matched controls. Collectively, we confirmed the *in* *silico* predictions in75% of patients using WB or IHC for CAPN3 levels/autolytic activity and RT-PCR for mRNA expression, including subject P12, but not in P4 or P10 (Fig. 1A–C; Table S1; Fig. S2). Immunoblotting was the most robust test in this study, while RT-PCR and IHC were less useful for diagnostic purposes. Gene sequencing was necessary for specific diagnosis in P4 and P10. Mutations predicted to be pathogenic are presented in Table 1 and novel variants are also described in the text.

Proband 1 is of European Canadian decent and diagnosed with classical pelvifemoral LGMD2A (Leyden-Möbius). *CAPN3* sequencing revealed a unique combination of missense mutations [c.1250 C>T (p.Thr417Met) and c.500 T>C (p.Phe167Ser)]. Upon biochemical analyses, moderate reductions in protein expression and autolytic activity of calpain-3 were noted, but no defects in mRNA abundance. The former sequence variant exchanges a highly conserved threonine for a methione in domain III and results in an absence of CAPN3 protein when combined with c.2362_2363delinsTCATCT [33]. The second mutation in proband 1 is novel and exchanges a highly conserved phenylalanine (nonpolar, neutral, and hydrophobic) for a serine (polar, neutral, and hydrophilic) in domain II, which contains the catalytic module of CAPN3. Because there is no biochemical data on either mutation in the homozygous state, individual contributions of each sequence variant to the phenotype are difficult to determine.

Proband 2 is of South-East Asian heritage and exhibited two compound heterozygous missense mutations [c.2338 G>C (p. Asp780His) and c.2366 T>A (p.Leu789Gln)] causing late-onset pelvifemoral LGMD2A. Protein expression and autolytic activity, but not mRNA levels, were significantly reduced and the biochemical phenotype was more severe than P1 despite the late-onset form. Both sequence variants exchange evolutionary conserved amino acids in domain IV of CAPN3 (exon 22), which may affect Ca^2+^-binding with downstream effects on autolytic activity. Interestingly, c.2338 G>C was inherited from his mother and is a recently recognized founder mutation originating from northern India in the Agarwal community, whose clan members practice intra-communal exogamy and are postulated to be descendants of King Agrasen [34]. This mutation was previously shown to cause pelvifemoral LGMD2A in the homozygous state and in combination with c.2099-1 G>T or c.1106 G>A, but effects on CAPN3 expression were inconclusive (protein non-significantly reduced vs. absent) [34], [35]. We speculate that the novel sequence variant c.2366 T>A has deleterious effects on protein expression and autolysis of CAPN3, although verification in a homozygous patient or through site-directed mutagenesis is preferred.

Proband 4 was born in Pakistan and died at 56 years of age due to respiratory insufficiency. The patient exhibited a classical pelvifemoral LGMD2A phenotype with an onset in his late teens and he was wheelchair-bound at the time of his death. We identified a novel homozygous transversion (c.2115+4 T>G) in intron 19, which is predicted to create a cryptic splice site that is used instead of the regular site. Activation of a previously dormant cryptic splice may result in exon skipping or intron retention, and considering that CAPN3 reactivity appeared largely normal on IHC in this patient, we surmise that the gene product is stable but that the protein is truncated or otherwise dysfunctional.

Proband 10 is of Irish decent and was clinically diagnosed with early onset pelvifemoral LGMD2A. The patient was found to be compound heterozygous for two missense mutations [c.1621 C>T (p.Arg541Trp) and c.338 T>C (p.Ile113Thr)], affecting conserved amino acids and predicted to be pathological by SIFT analysis. While the muscle biopsy showed a definite dystrophic pattern, we did not detect CAPN3 deficiency using the standard RT-PCR, WB, or IHC tests, possibly indicating that other functions, such as substrate recognition/binding or proteolytic activity, are impaired. c.1621 C>T has previously been reported in LGMD2A patients in the compound heterozygous and homozygous states and is associated with a reduction in CAPN3 expression in both Erb and Leyden-Möbius phenotypes [13], [36]. c.338 T>C is a novel mutation exchanging isoleucine (nonpolar, neutral, and hydrophobic) for threonine (polar, neutral and hydrophilic), and is predicted by SIFT to be pathogenic and may impair proteolytic activity in domain II of CAPN3.

Proband 12 is of Iranian heritage and exhibited the major diagnostic criteria for primary calpainopathy, but clinical records were insufficient to pinpoint a specific LGMD2A phenotype. The mutational screen revealed a novel transversion in intron 11 (c.1525-1 G>T) that was predicted to destroy an acceptor splice site, but a second sequence variant was not identified. Canonical mutations of conserved nucleotides in positions −2, −1, +1, and +2 will significantly affect splicing (minus/plus signs indicate upstream/downstream from 5′ and 3′ ends of the exon, respectively) and may cause exon skipping or intron retention [37], ultimately affecting the mRNA stability and/or protein structure. CAPN3 protein levels, mRNA abundance, and autolytic activity were significantly reduced in P12, which strongly suggests that the mutation is pathogenic and confirms the clinical diagnosis.

### Redox balance and mitochondrial function

**Figure 2:**
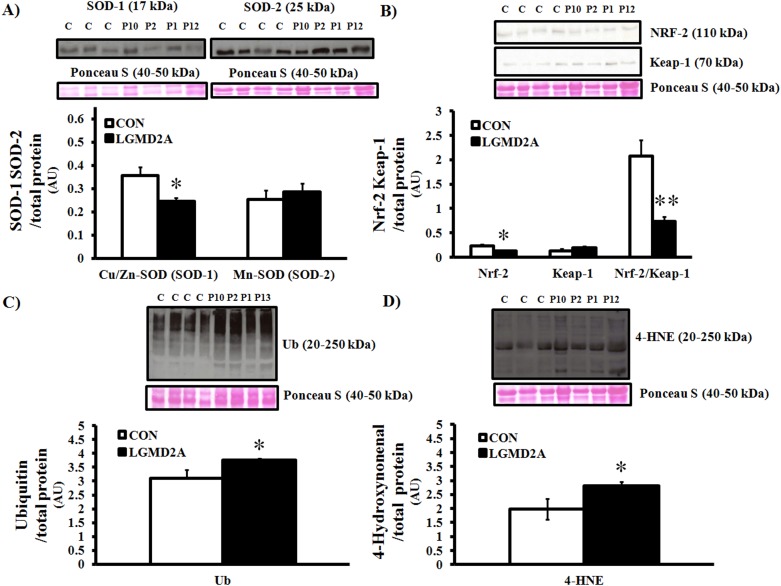
Western blot analyses of anti-oxidant capacity (SOD-1, SOD-2, NRF-2/Keap-1), oxidative damage (lipid peroxidation; 4-HNE), and ubiquitination (Ub) in LGMD2A patients. *P≤0.05 vs. control. **P≤0.01 vs. control. All data were normalized to total protein levels (Ponceau S stain) and represent averages of age/gender-matched controls (N = 3–4; white bars) and LGMD2A patients (N = 4; black bars). Representative images of blots and Ponceau S stain (40–50 kDa) are shown.

**Figure 3:**
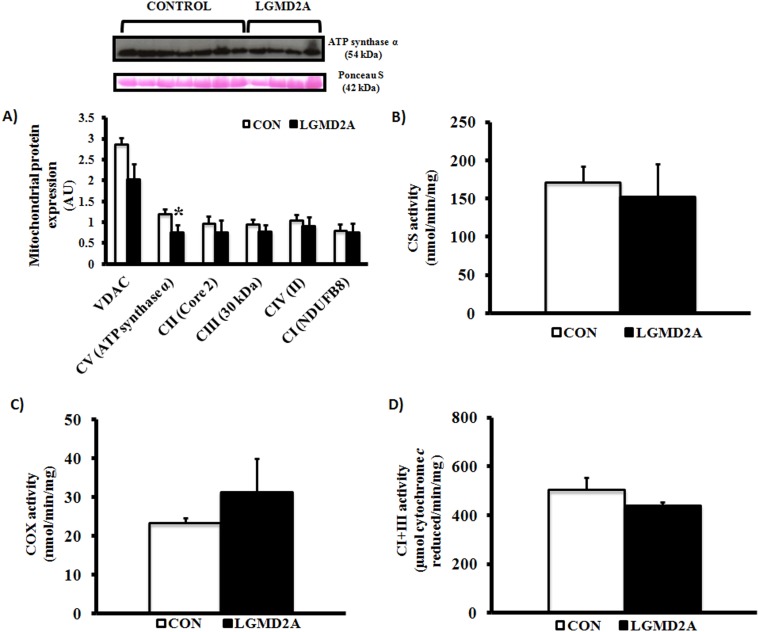
Western blot analyses of OXPHOS expression (complex I–V) and ETC enzyme activities (CS, COX, and complex I+III) in LGMD2A patients. *Significantly lower ATP synthase expression in calpain-3 deficient muscle (P≤0.05). All Western blot data were normalized to total protein levels (Ponceau S stain) and represent averages of age/gender-matched controls (N = 7 for OXPHOS and enzyme assays; N = 5 for VDAC) and LGMD2A patients (N = 5 for OXPHOS and VDAC; N = 2–3 for enzyme assays). Representative images of OXPHOS blots and Ponceau S stain (42 kDa) are shown.

Although structural and distributional abnormalities of mitochondria, oxidative damage, and markers of degradation may be hallmarks of the dystrophic process in calpain-3 deficient skeletal muscle [18], [20], [38], mitochondrial enzyme function and anti-oxidant defense have not been assessed in LGMD2A patients to date. As such, we measured the expression of CuZn-SOD (SOD-1), Mn-SOD (SOD-2), Nrf-2/Keap-1 and assessed the degree of oxidative damage, protein ubiquitinylation, and mitochondrial function in a sub-set of patient biopsies. Interestingly, SOD-1 and the Nrf-2/Keap-1 ratio were significantly lower in LGMD2A tissue, while SOD-2 levels were normal (Fig. 2A–B). As expected, lipid peroxidation (4-HNE) and total ubiquitin expression were elevated in calpain-3 deficient muscle (Fig. 2C–D). Activities of citrate synthase, COX, and complex I+III were not different from age/gender-matched controls, although a moderate reduction of complex V (ATP synthase) was detected on immunoblots (Fig. 3A–D).

## Discussion

Herein we present five novel sequence variants that add to the mutational spectrum of LGMD2A and provide further insights into the cellular mechanisms underlying pathology in this patient population. Our results support the contention that gel-based tests (protein expression and autolytic activity) are the most important diagnostic tools next to *CAPN3* gene sequencing, while microscopy-based methods may be less useful for genotype-phenotype correlations and confirmation of clinical diagnosis.

In addition to our mutational data, we assessed oxidative damage, key anti-oxidant markers, and mitochondrial enzyme function in a subset of patient biopsies. Although the limited sample size prohibits us from making generalized statements, our findings leave open the possibility of mitochondrial enzyme dysfunction not being a universal feature of calpainopathy in humans, which appears to be the case in CAPN3 KO mice [16]. We found that the activities of rate-limiting enzymes in the Kreb’s cycle (citrate synthase), mitochondrial respiratory chain (cytochrome *c* oxidase and NADH-cytochrome *c* oxidoreductase), and expression of OXPHOS proteins were largely normal in LGMD2A patients. In support of these observations, SOD-2 levels were comparable to age- and gender-matched controls, suggesting a preserved ability of mitochondria to catalyze highly reactive superoxide anions from complex I and III into hydrogen peroxide. Conversely, Nrf-2, Nrf-2/Keap1 ratio, and CuZn-SOD (SOD-1) were significantly suppressed in calpain-3 deficient skeletal muscle, collectively indicating a primary cytosolic/myofibrillar redox imbalance. Considering that Nrf-2 dissociates from its cytosolic inhibitor Keap1 and moves to the nucleus to regulate the transcription of anti-oxidant genes under stress conditions, aberrant regulation of Nrf-2 is generally synonymous with a reduction in cytoprotection, as previously shown by our group [39]. Given the results of the current study, and others [20], [40], [41], cellular redox imbalance appears to be compartment-specific and induces the ubiquitin-proteasome pathway, which in turn orchestrates protein degradation and muscle wasting in LGMD2A.

Calpain-3 associates with the ryanodine receptor and regulates Ca^2+^ release at the muscle triads, and calpain-3 deficiency impairs Ca^2+^ transport, RyR expression, and CAMKII signaling in CAPN3 KO mice [6], [7], [8]. Calcium kinetics play an important role in loading-induced muscle adaptations and maintenance of slow fiber phenotype in humans, and LGMD2A patients may exhibit lower RyR levels and a preferential involvement of slow muscle fibers [7]. Although the connection between calpain-3 and mitochondrial function remains unclear, two reports have demonstrated mitochondrial abnormalities in Japanese LGMD2A patients [18], [38]. In partial agreement with aforementioned studies, ATP synthase levels were suppressed and type 1 fibers preferentially affected in our patient cohort, but we did not find general deficits in mitochondrial enzyme function, protein expression, or ultra-structure. Interestingly, ATP production is impaired in CAPN3 KO mice [16], but neither ATP synthase activity or energy status were assessed in our study. Because Ca^2+^ modulates the phosphorylation of subunit *c* of F_0_F_1_ ATPase and increased Ca^2+^ levels activates a number of nuclear genes encoding mitochondrial proteins (including ATP synthase) [42], [43], calpainopathy may impair transcription of ATP synthase and/or its function. Selective type 1 fiber atrophy conceivably lowers the total amount of mitochondria in CAPN3-deficient skeletal muscle, and in light of our finding that VDAC was symmetrically reduced (albeit not significantly) compared to F_0_F_1_ ATPase, further research is necessary to delineate potential complex-specific deficiencies in isolated mitochondrial fractions.

In summary, we confirmed pathogenicity of *CAPN3* mutations in 75% of LGMD2A patients selected for biochemical analyses, which stresses the importance of gene sequencing in the diagnostic process. Assessment of total CAPN3 expression and autolysis by immunoblotting were the most reliable diagnostic tools (compared to RT-PCR and IHC) in the absence of substrate-specific enzymatic activity assays. We confirmed that oxidative damage is a hallmark of LGMD2A and expanded on previous studies by showing that calpain-3 deficiency is associated with cytosolic redox imbalance and a mild reduction in ATP synthase levels. Given the fact that CAPN3 regulates calcium release and Ca^2+^ is necessary for transcription and function of ATP synthase, future studies elucidating potential deficits in ATP production and F_0_F_1_ ATPase activity in LGMD2A patients may be warranted.

## Supporting Information

Figure S1 **Localization and distribution of 21** ***CAPN3*** **sequence variants from our cohort of 14 LGMD2A patients along pre-mRNA (top), mRNA (middle), and protein (bottom).** Mutational data adhere to the guidelines proposed by the Human Genome Variation Society (www.hgvs.org/mutnomen) and nucleotide numbering reflects the cDNA sequence with +1 corresponding to the A of the ATG translation initiation codon in the reference sequence. Exons 4, 11, 13, and 22 show the highest number of mutations, affecting protein domains II (3 mutations), III (4 mutations), and IV (2 mutations). Intronic mutations mainly affect domains III and IV. Domain I has regulatory role, domain II is the proteolytic module, domain III has a C2-like domain, and domain IV binds Ca^2+^ ions. NS, IS1, and IS2 are calpain-3 specific insertions.
(TIF)

Figure S2 **Hematoxylin and eosin stain, immunohistochemical determination of fiber-type, and CAPN3 reactivity on flat sections in a sub-set of LGMD2A patients (total magnification 200×−400×).** Top panel: Red circles show markedly increased abundance of internalized nuclei in calpain-deficient muscle. Middle panel: Primarily slow-twitch fiber atrophy and increased variation in fiber size/shape in LGMD2A patients. Note fast-twitch fiber dominance in P11. Lower panel: Normal CAPN3 reactivity in all subjects despite pathological mutations.
(TIF)

Table S1 ^a^Clinical diagnosis: Phenotype consistent with major criteria of LGMD2A (see methods); ^b^CAPN3 mRNA expression by RT-PCR (Fig. 1A); ^c^Total CAPN3 protein expression on Western blot (Fig. 1B); ^d^Ca^2+^-induced CAPN3 autolytic activity assessed on immunoblot (Fig. 1C); ^e^CAPN3 reactivity on 8 µm frozen sections (Fig. S2); ND: Not done; INDT: Indeterminate.
(DOC)
