# Supplementary figures and images for: Redox State and Mitochondrial Respiratory Chain Function in Skeletal Muscle of LGMD2A Patients
Source: PLoS One. 2014 Jul 31;9(7):e102549. doi: 10.1371/journal.pone.0102549 (PMC4117472; doi:10.1371/journal.pone.0102549)

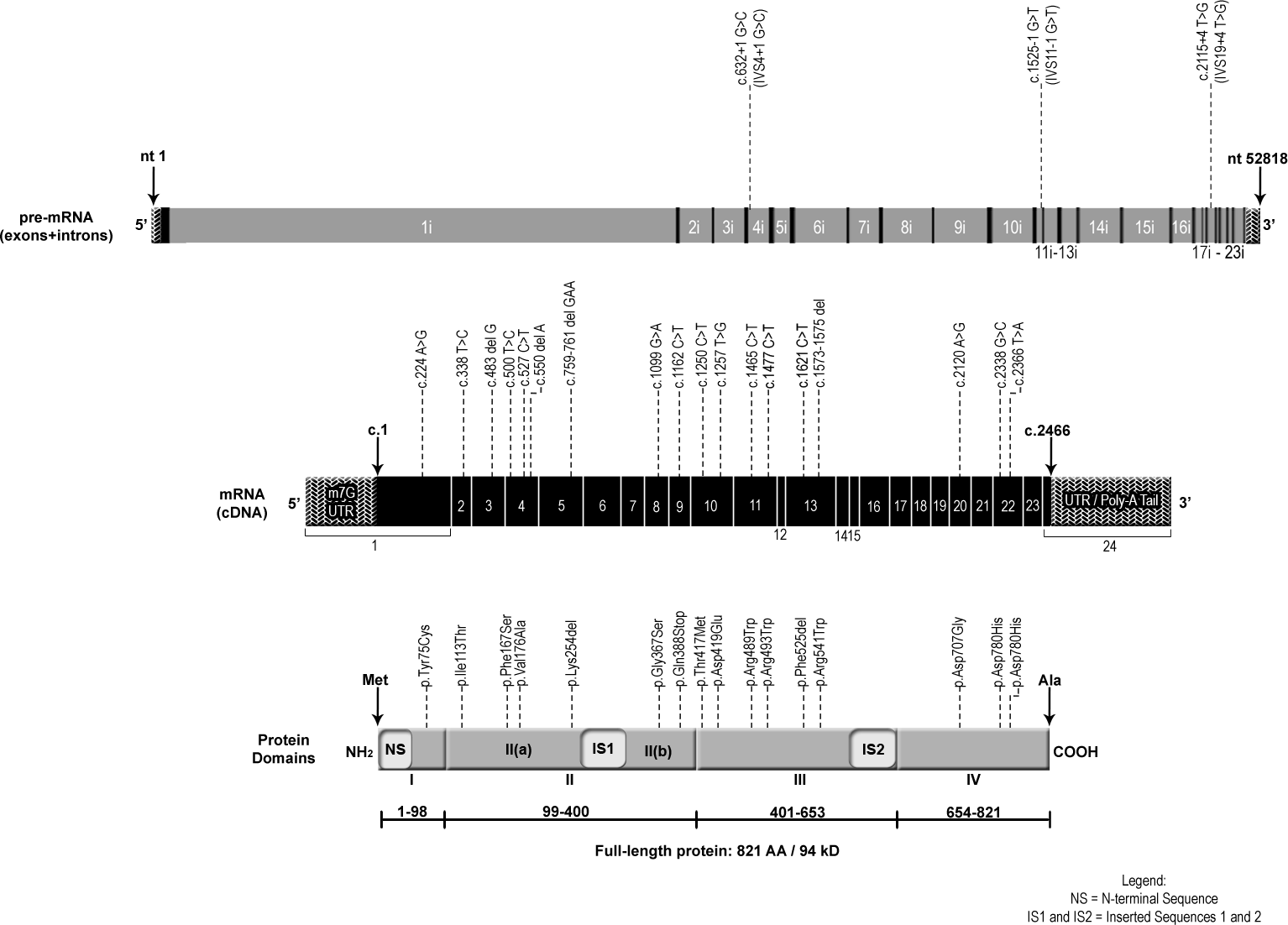

Supplement: Figure S1 — Localization and distribution of 21 CAPN3 sequence variants from our cohort of 14 LGMD2A patients along pre-mRNA (top), mRNA (middle), and protein (bottom). Mutational data adhere to the guidelines proposed by the Human Genome Variation Society (www.hgvs.org/mutnomen) and nucleotide numbering reflects the cDNA sequence with +1 corresponding to the A of the ATG translation initiation codon in the reference sequence. Exons 4, 11, 13, and 22 show the highest number of mutations, affecting protein domains II (3 mutations), III (4 mutations), and IV (2 mutations). Intronic mutations mainly affect domains III and IV. Domain I has regulatory role, domain II is the proteolytic module, domain III has a C2-like domain, and domain IV binds Ca2+ ions. NS, IS1, and IS2 are calpain-3 specific insertions. (TIF) [file pone.0102549.s001.tif]

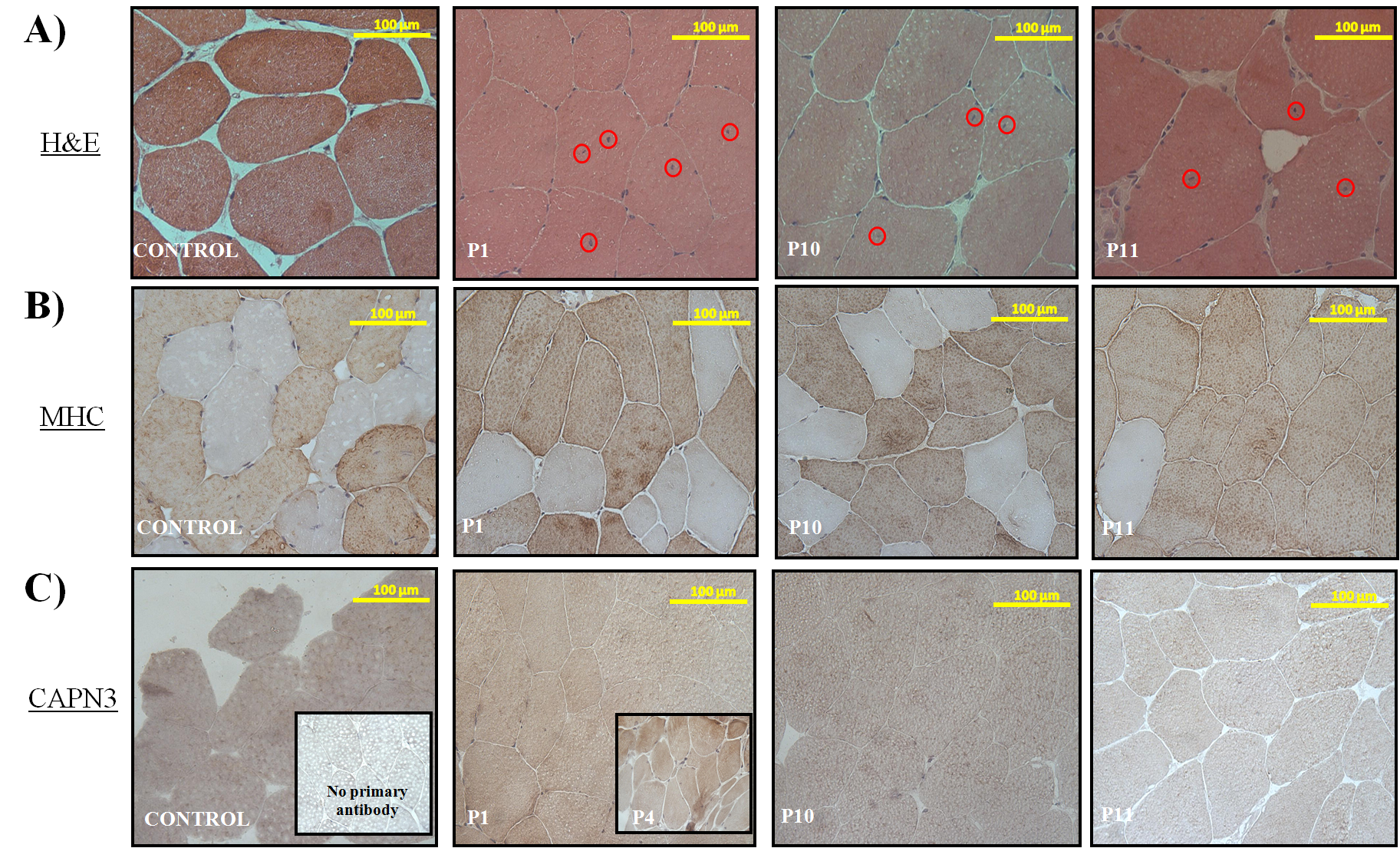

Supplement: Figure S2 — Hematoxylin and eosin stain, immunohistochemical determination of fiber-type, and CAPN3 reactivity on flat sections in a sub-set of LGMD2A patients (total magnification 200×−400×). Top panel: Red circles show markedly increased abundance of internalized nuclei in calpain-deficient muscle. Middle panel: Primarily slow-twitch fiber atrophy and increased variation in fiber size/shape in LGMD2A patients. Note fast-twitch fiber dominance in P11. Lower panel: Normal CAPN3 reactivity in all subjects despite pathological mutations. (TIF) [file pone.0102549.s002.tif]
