# Supplementary material for: Redox State and Mitochondrial Respiratory Chain Function in Skeletal Muscle of LGMD2A Patients
Source: PLoS One. 2014 Jul 31;9(7):e102549. doi: 10.1371/journal.pone.0102549 (PMC4117472; doi:10.1371/journal.pone.0102549)
Supplement: Table S1 — aClinical diagnosis: Phenotype consistent with major criteria of LGMD2A (see methods); bCAPN3 mRNA expression by RT-PCR (Fig. 1A); cTotal CAPN3 protein expression on Western blot (Fig. 1B); dCa2+-induced CAPN3 autolytic activity assessed on immunoblot (Fig. 1C); eCAPN3 reactivity on 8 µm frozen sections (Fig. S2); ND: Not done; INDT: Indeterminate. (DOC) [file pone.0102549.s003.doc]

**Table 2. Clinical and biochemical diagnosis of select LGMD2A patients.**

| **ID** | **^a^Clinical**  **diagnosis** | **^b^CAPN3 mRNA**  **(Transcript variant 1)**  **% CON** | **^b^CAPN3 WB**  **(Autolyzed + full-length)**  **% CON** | **^d^CAPN3 autolysis**  **(Autolyzed/Total CAPN3)**  **% CON** | **^e^CAPN3 IHC** |
| --- | --- | --- | --- | --- | --- |
| **P1** | Positive | +26% | -40% | -22% | Expressed |
| **P2** | Positive | ND | -60% | -99% | ND |
| **P3** | Positive | ND | -69% | INDT. | ND |
| **P4** | Positive | ND | ND | ND | Expressed |
| **P10** | Positive | +55% | -8% | +10% | Expressed |
| **P11** | Positive | -23% | -28% | +10% | Expressed |
| **P12** | Positive | -39% | -58% | -37% | ND |
| **P13** | Positive | ND | -68% | -84% | ND |

^a^Clinical diagnosis: Phenotype consistent with major criteria of LGMD2A (see methods);^b^CAPN3 mRNA expression by rtPCR (Fig. 2A); ^c^Total CAPN3 protein expression on Western blot (Fig. 2B); ^d^Ca^2+^-induced CAPN3 autolytic activity assessed on immunoblot (Fig. 2C); ^e^CAPN3 reactivity on 8 µm frozen sections (Supplemental Fig. 1); ND: Not done; INDT: Indeterminate.
